# Identification of *Campylobacter jejuni* and *Campylobacter coli* genes contributing to oxidative stress response using TraDIS analysis

**DOI:** 10.1186/s12866-024-03201-y

**Published:** 2024-02-01

**Authors:** Emily Stoakes, Xuanlin Chen, Lajos Kalmar, Dave Baker, Rhiannon Evans, Steven Rudder, Andrew J. Grant

**Affiliations:** 1 Department of Veterinary Medicine, University of Cambridge, Madingley Road, Cambridge, UK; 2 MRC Toxicology Unit, University of Cambridge, Tennis Court Road, Cambridge, UK; 3 Quadram Institute Bioscience, Norwich Research Park, Norwich, UK

**Keywords:** *Campylobacter jejuni*, *Campylobacter coli*, TraDIS, Oxidative stress, Aerobic stress

## Abstract

**Background:**

*Campylobacter jejuni* and *Campylobacter coli* are the major causative agents of bacterial gastroenteritis worldwide and are known obligate microaerophiles. Despite being sensitive to oxygen and its reduction products, both species are readily isolated from animal food products kept under atmospheric conditions where they face high oxygen tension levels.

**Results:**

In this study, Transposon Directed Insertion-site Sequencing (TraDIS) was used to investigate the ability of one *C. jejuni* strain and two *C. coli* strains to overcome oxidative stress, using H_2_O_2_ to mimic oxidative stress. Genes were identified that were required for oxidative stress resistance for each individual strain but also allowed a comparison across the three strains. Mutations in the *perR* and *ahpC* genes were found to increase *Campylobacter* tolerance to H_2_O_2_. The roles of these proteins in oxidative stress were previously known in *C. jejuni*, but this data indicates that they most likely play a similar role in *C. coli*. Mutation of *czcD* decreased *Campylobacter* tolerance to H_2_O_2_. The role of CzcD, which functions as a zinc exporter, has not previously been linked to oxidative stress. The TraDIS data was confirmed using defined deletions of *perR* and *czcD* in *C. coli* 15-537360.

**Conclusions:**

This is the first study to investigate gene fitness in both *C. jejuni* and *C. coli* under oxidative stress conditions and highlights both similar roles for certain genes for both species and highlights other genes that have a role under oxidative stress.

**Supplementary Information:**

The online version contains supplementary material available at 10.1186/s12866-024-03201-y.

## Background

*Campylobacter* species are the leading cause of human bacterial gastroenteritis worldwide. Within the UK, *Campylobacter jejuni* causes ~ 90% of campylobacteriosis cases, whilst *Campylobacter coli* causes ~ 10% of cases and overall cases cost the economy ~£0.71 billion per annum [1, 2]. Reservoirs of *Campylobacter* include undercooked meat such as poultry, pork and beef. The majority of UK *C. jejuni* cases are attributed to chickens. Although *C. coli* is also linked to chickens it is the dominant *Campylobacter* spp. in pigs [3]. *Campylobacter* species have fastidious growth requirements. Their optimal conditions include temperatures between 37 and 42 °C with low levels of oxygen. They are often described as being sensitive to osmotic stress, desiccation, low pH levels and high temperatures [4].

Both *C. jejuni* and *C. coli* have been found to grow optimally at partial oxygen tension of 2–10%, however they inevitably must survive in atmospheres with a partial oxygen tension of 21% when they are excreted from humans and other animals [5]. Therefore, the ability to overcome oxidative stress and aerobic stress is critical for persistence in the food chain and the subsequent transmission between animals and humans [6].

Exposure to oxidative stress means the generation of reactive oxygen species (ROS) is unavoidable. *C. jejuni* and *C. coli* both possess several regulators such as PerR (peroxide resistance regulator), Fur (ferric uptake regulator) and CosR (*Campylobacter* oxidative stress regulator) that co-ordinate expression of genes whose protein products are important for surviving oxidative stress [7, 8]. Previous studies have identified that several genes belonging to the PerR regulon have potential functions in oxidative stress defense [8]. PerR negatively regulates the transcription of superoxide and peroxide resistance genes in an iron-dependent manner [7]. Transcription of *perR* is auto-regulated and its regulatory role in oxidative stress defence is induced by conformational changes in PerR [9]. The exact sensing and regulatory mechanisms of PerR remain to be elucidated. PerR and Fur proteins have some level of overlap in regulating antioxidant proteins [8]. CosR also regulates the expression of antioxidants involved in superoxide and peroxide defence, whereas it is specifically responsive to superoxide, but not to H_2_O_2_ [10]. In addition to these regulators that are commonly found in both *C. jejuni* and *C. coli*, *C. jejuni* also possesses unique transcriptional regulators RrpA and RrpB (regulator of response to peroxide) for oxidative stress defense [11, 12].

Both *C. jejuni* and *C. coli* have a range of antioxidants, such as SodB, KatA, AhpC, Bcp, Tpx and two cytochrome c peroxidases that also are involved in ROS detoxification. SodB (superoxide dismutase) removes superoxide stress by catalysing the conversion of O^2−^ to oxygen and the relatively weakly reactive H_2_O_2_ [13]. Whilst the most well-characterised H_2_O_2_ scavenger, KatA (catalase), is induced by both H_2_O_2_ and O^2−^ and reduces H_2_O_2_ into water and oxygen [13, 14]. AhpC, Bcp and Tpx are peroxiredoxins that convert H_2_O_2_ and organic peroxides into water and alcohols [15–17]. At low oxygen concentrations, AhpC (alkyl hydroperoxide reductase) is believed to be the predominant H_2_O_2_ scavenger [18]. Insertional mutation of *ahpC* leads to the build-up of ROS within the bacterial cells and mutants are hypersensitive to cumene hydroperoxide (an organic peroxide), whereas the resistance to H_2_O_2_ was not affected by the *ahpC* mutation [15]. The peroxiredoxin family in *C. jejuni* also includes two putative peroxidases, thiol peroxidase (Tpx) and bacterioferritin comigratory protein (Bcp); Tpx plays a major role in cellular H_2_O_2_ detoxification [17].

Previous genome-wide studies into *C. jejuni* oxidative stress have employed targeted mutant studies and transcriptomic or proteomic approaches to identify genes involved in oxidative stress defence [8, 19, 20]. Similar studies have not yet been conducted in *C. coli.* Other high-throughput technologies such as TraDIS (Transposon Directed Insertion-site Sequencing) combines transposon (Tn) mutagenesis with Next Generation DNA sequencing and allows a direct link between observed phenotype and genotype [21]. Through the creation of a large pool of mutants using a Tn to disrupt genes and the identification of its position in the genome it is possible to find if the insertion of the Tn increases or decreases the fitness of the bacteria under defined conditions [21]. The molecular mechanisms underlying the survival strategies of *Campylobacter* spp, particularly *C. jejuni* and *C. coli*, under oxidative stress in the food chain are not fully understood. The generation of comprehensive Tn-insertion mutant libraries in both *C. jejuni* and *C. coli* [22] means that high throughput analysis of genes required for oxidative stress response can be completed.

In this study, *C. jejuni* and *C. coli* Tn-insertion mutant libraries were exposed to H_2_O_2_ to assess genes required for survival under oxidative stress. To the best of our knowledge, this is the first published Tn mutagenesis study within *C. coli* and the analysis re-confirms roles of previously known oxidative stress proteins for *C. jejuni.* We also found genes whose proteins have not been linked to the oxidative stress response before for both *C. jejuni* and *C. coli.* The identification of key determinants that are functionally important for stress response and adaptation might lead to improved strategies aimed at reducing *Campylobacter* in the food chain.

## Results

### Survival assay of strains in response to varying concentrations of H_2_O_2_

**Fig. 1:**
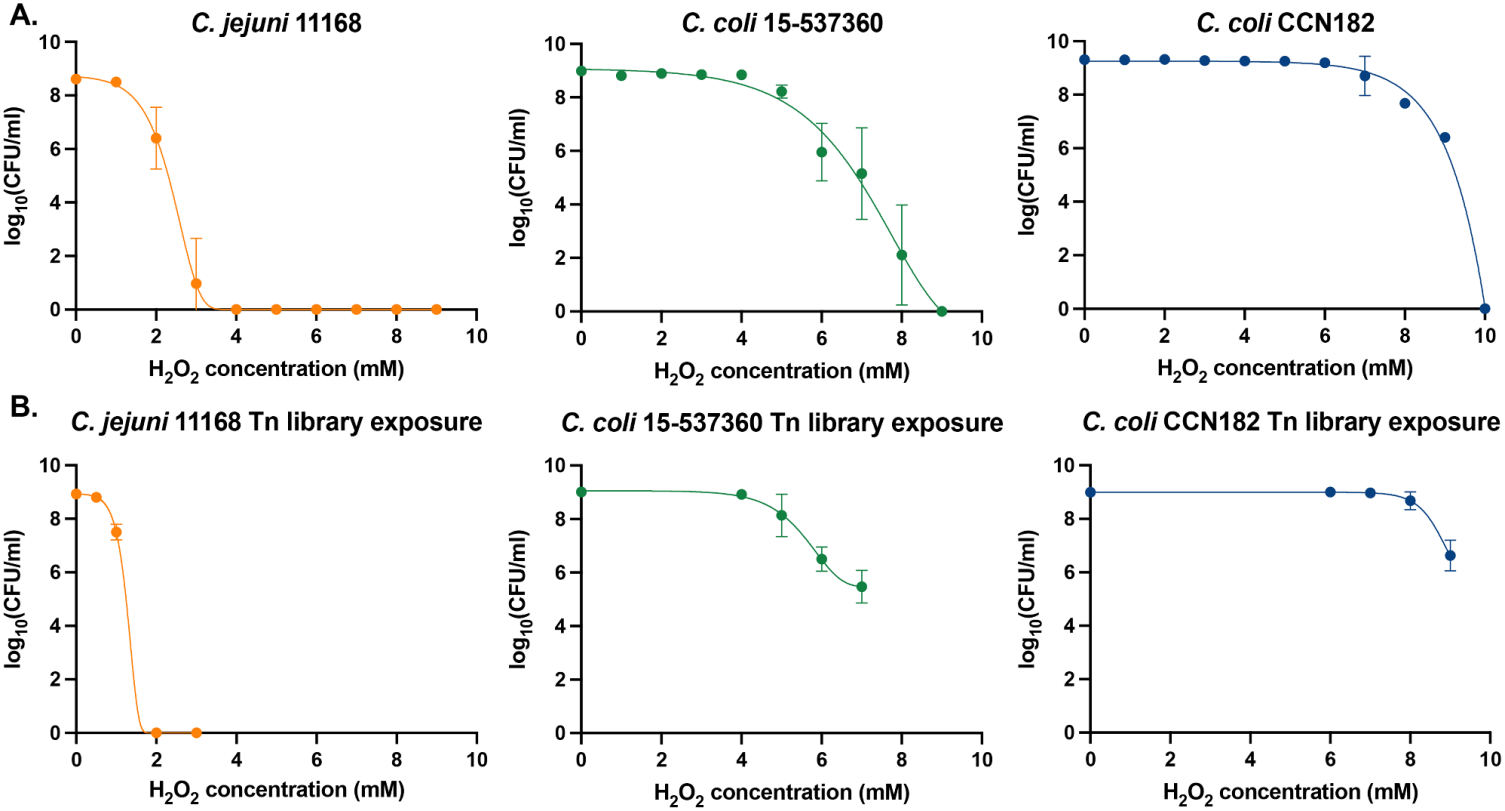
Survival of *Campylobacter* strains after exposure to various concentrations of H_2_O_2_. The mean Log_10_(CFU/mL) and standard deviation plotted against increasing H_2_O_2_ concentrations after incubation for 16 h (*N*≥3). Graphs show each wild-type strain (**A**) and the Tn-insertion mutant library for each strain (**B**) after exposure to varying concentrations of H_2_O_2_

The effect of H_2_O_2_ varied across the three *Campylobacter* isolates. For example, the survival of *C. jejuni* 11168 started to decrease from 1 to 2mM H_2_O_2_ and by 4mM H_2_O_2_ there was no survival, whereas both *C. coli* strains exhibited no effects until higher concentrations of H_2_O_2_ (Fig. 1A). For *C. coli* 15-537360, until 5mM H_2_O_2_ there was no noticeable decrease in survival and there was complete cell death at 9mM H_2_O_2_ (Fig. 1A). Survival of *C. coli* CCN182 started to decrease at 7mM H_2_O_2_ and had complete cell death by 10mM H_2_O_2_.

### Results of the H_2_O_2_ TraDIS screen

For the H_2_O_2_ TraDIS screen, a series of H_2_O_2_ concentrations were selected to expose the pool of Tn-insertion mutants to (Fig. 1B). For *C. jejuni* 11168 there was a difference in the survival of the Tn-insertion mutants compared to the wild-type, with the Tn-insertion mutant pool dying off completely by 2mM. This may be due to competition and/or compensation within the pool of Tn-insertion mutants. After performing the exposure, concentrations were selected to achieve ~ 60–80% survival of each Tn-insertion mutant library. The Tn-insertion mutant libraries exposed to the following concentrations were sequenced: 1mM for *C. jejuni* 11168, 6mM for *C. coli* 15-537360 and 9mM for *C. coli* CCN182. In order to identify the number of Tn-insertion mutants that were either detrimental (and therefore under-represented in the library) or provided a gain-of-function (and therefore over-represented in the library) when exposed to H_2_O_2_, the outputs from these concentrations were compared to the same libraries under the same conditions that were only exposed to water (Table 1, Supplementary File 1).

**Table 1:** Number of over- or under-represented Tn-insertion mutants for each strain after exposure to H_2_O_2_

Species and Strain	No. of over-represented Tn-insertion mutants	No. of under-represented Tn-insertion mutants
*C. jejuni* 11168	25 (1)	10
*C. coli* 15-537360	5	143 (3)
*C. coli* CCN182	4	52

Over-represented and under-represented Tn-insertion mutants were determined using a Log_2_ fold change of 2 or -2, respectively, and a q value of less than 0.05. Numbers in parenthesis indicate the number of pseudogenes that were significantly highlighted as under- or over-represented Tn-insertion mutants. Complete lists of genes may be found in Supplementary File 1.xls

### H_2_O_2_ response in *C. jejuni* 11168

For *C. jejuni* 11168, there were 26 Tn-insertion mutants that were over-represented under oxidative stress, indicating that mutating these genes enhanced tolerance to oxidative stress. Tn-insertion mutants in the gene *perR*, which codes for the peroxide stress regulator, and is known to negatively regulate transcription of oxidative stress regulated genes, had the largest Log_2_fold change when disrupted. Tn-insertion mutants in genes which encode for iron acquisition and storage also were found to have increased oxidative stress tolerance, such as ferritin, and *cfbpAB*. Transposon-insertion mutants in *ahpC*, an alkyl hydroperoxide reductase, which is known to be regulated in response to iron, were also found to be over-represented after exposure. Transposon disruption in the Ni/Fe hydrogenase (*hydABC*) also provided increased tolerance. Several Tn-insertion mutations in genes encoding transferases and transporters also had a gain-of-function role, such as Cj1584c, Cj0622, Cj1432c, Cj1684c. Transposon-insertion mutants in *flaA* and *cheW*, both involved in motility also were over-represented. There were two hypothetical proteins, Cj1009c and Cj0808c whose disruption provided a protective effect against oxidative stress but to the best of our knowledge have not been found before in a H_2_O_2_ screen. Cj1009c shows homology to the C- terminal domain of *E. coli* TrkA, a potassium ion channel but has not been experimentally investigated [23, 24]. Cj0808c, according to Interpro has domains both predicted to be embedded in the membrane and in the cytoplasm [25].

There were 10 Tn-insertion mutants that were under-represented indicating that their disruption caused a decreased tolerance to oxidative stress. Transposon-insertion mutants in the gene encoding Cj1535c, a bacterioferritin, had the greatest Log_2_fold decrease of -7.68, followed by the gene encoding Cj0818, a lipoprotein. Disruption of a gene encoding another iron-related protein, an iron permease (Cj1658), were under-represented after exposure to H_2_O_2_. Tn-insertion mutants in the gene encoding CzcD (Cj1163c), a cation transporter was also found to be disadvantageous. Similarly, disruption to the zinc transporter, *zupT*, also had a similar effect. Transposon-insertion mutants in *tpX*, a thiol peroxidase which is known to catalyse the breakdown of H_2_O_2_ into water and alcohol, were attenuated for survival after exposure.

### H_2_O_2_ response in *C. coli* 15-537360 and CCN182

For *C. coli*, unlike *C. jejuni*, there were more Tn-insertion mutants that caused a decrease than an increase in tolerance to oxidative stress. For those that caused an increase in tolerance, there were only 5 (*C. coli* 15-537360) and 4 (*C. coli* CCN182) Tn-insertion mutants that had an advantageous effect. For *C. coli* 15-537360 Tn-insertion mutants in *perR* and *ahpC* had the greatest Log_2_fold increase after H_2_O_2_ exposure. Transposon-insertion mutants in a *cheY*-like response regulator and a NCS2 family permease (N149_1061, N149_1333) also resulted in increased tolerance. Transposon-insertions in a gene encoding a hypothetical protein, N149_0152, also were found to be advantageous. For *C. coli* CCN182, Tn-insertion mutants in *perR* and *ahpC* were also found to increase tolerance to oxidative stress. Transposon-insertions into two genes encoding hypothetical proteins were also found to increase tolerance. One of these was annotated by Bakta to be a flagellar biosynthesis protein, however, further investigation of the literature and BlastP/InterPro could not define a role for this protein in *Campylobacter* except for it being a membrane protein. The other was annotated as hypothetical, and no functional domains were identified using InterPro [25].

In *C. coli* 15-537360, Tn-insertion mutants in several known oxidative stress response genes, such as *sodB*, *katA*, and a thiol peroxidase (N149_0720) were under-represented. Transposon-insertion mutations in several flagellar and chemotaxis genes also caused a decrease in fitness (*flaA, flgH, flgI, fliD*, *cheB*). Transposon-insertion mutants in 20 genes annotated as encoding transporters were also found to be involved in resistance to oxidative stress. These included two ABC transporter permease subunits (N149_0465, N149_0892), two iron transporters (N149_1113 (iron transporter), N149_1315 (iron chelate uptake ABC transporter subunit), *cmeEF* which form part of an RND-type efflux pump and several genes involved in methionine (N149_0712), serine/threonine (*sstT*), molybdate (*modB*), arsenic (N149_1127), and aspartate/glutamate transportation (*peb1A*). We identified that Tn-insertion mutations in two cation transporters (N149_1105, N149_1635) were under-represented after H_2_O_2_ exposure.

In *C. coli* CCN182, Tn-insertions in a few genes whose proteins are involved in stress response were identified to be involved in the response to H_2_O_2_ exposure, such as ribosomal silencing factor (*rsfS*) and the *uvrABC* system protein C which performs part of the excision repair mechanism for DNA. Transposon-insertions in genes encoding proteins that suggest a role in oxidative stress protection were also found to be disadvantageous - a thioredoxin, nitroreductase family protein and a NAD(P)H-dependent oxidoreductase. Similarly, to *C. coli* 15-537360, Tn-insertions in a flagellar gene caused a decrease in fitness, *flgQ*, a gene whose protein product is required for the stability and localization of the FlgP protein to the outer membrane was also found. Like *C. coli* 15-537360, Tn-insertion mutations in transporters were found to be detrimental to *C. coli* CCN182 after exposure to H_2_O_2_. Transposon-insertion mutants in seven transporters including ones involving heme and methionine were found (ccn182_09045, ccn182_00505) to have a detrimental effect in survival under H_2_O_2_. Transposon-insertion mutation in the gene encoding the AraC transcriptional regulator was also found to be disadvantageous under H_2_O_2_ exposure.

For *C. coli* 15-537360 and *C. coli* CCN182, there were many Tn-insertion mutants in genes encoding proteins with unknown functions that were under-represented compared to the control. We identified 21 genes (16% of all under-represented Tn-insertion mutants) and 12 genes (23% of all under-represented Tn-insertion mutants) whose disruption caused a disadvantageous effect under oxidative stress that were annotated as uncharacterized proteins for *C. coli* 15-537360 and CCN182, respectively.

### Under or over-represented Tn-insertion mutants in pseudogenes

For both *C. jejuni* 11168 and *C. coli* 15-537360, there were Tn-insertion mutants in pseudogenes that were significantly over- or under-represented under oxidative stress conditions and therefore appear to have an advantageous or disadvantageous affect under H_2_O_2_ despite not coding a full-length functioning protein. Tn-insertion mutants in the Cj0072c pseudogene in *C. jejuni* 11168 were over-represented and therefore mutants in this are deemed advantageous under 1mM H_2_O_2_. This pseudogene encodes a putative iron-binding protein. In *C. coli* 15-536370, Tn-insertion mutants in three pseudogenes were under-represented, and therefore these mutants are disadvantageous under 6mM H_2_O_2_ exposure. The Tn-insertion mutants were in N149_1002, N149_1740 and N149_1175. N149_1175 is annotated as an AgrC accessory regulator, which in other bacteria (such as *Staphylococcus aureus*) has been found to be the sensor kinase of the Agr quorum sensing system that mediates bacterial oxidation [26]. N149_01740 is annotated as a bacteriohemerythrin. In *C. jejuni*, other hemerythrins have been found to protect iron-sulphur enzymes from oxidative damage [23]. N149_1002 is annotated as a NAD(P)H-dependent oxidoreductase.

Tn insertions were manually inspected to ensure that insertions were distributed across the pseudogene, and that the insertion counts (number of sequence reads) before and after H_2_O_2_ exposure did increase (Cj0072c) or decrease (N149_1002, N149_1740 and N149_1175). The distribution of Tn insertions were checked ensuring that the significance of the Tn-insertion mutants was not due to a portion of the pseudogene being functional or the potential presence of an sRNA. Having confirmed this, the sequence of all four pseudogenes was checked by Sanger sequencing. No changes were found when compared to the reference, indicating that in both strains, that at least at the DNA level, these genes were indeed pseudogenes.

### Comparison of genes involved in H_2_O_2_ between *C. jejuni* 11168, *C. coli* 15-537360 and *C. coli* CCN182

**Fig. 2:**
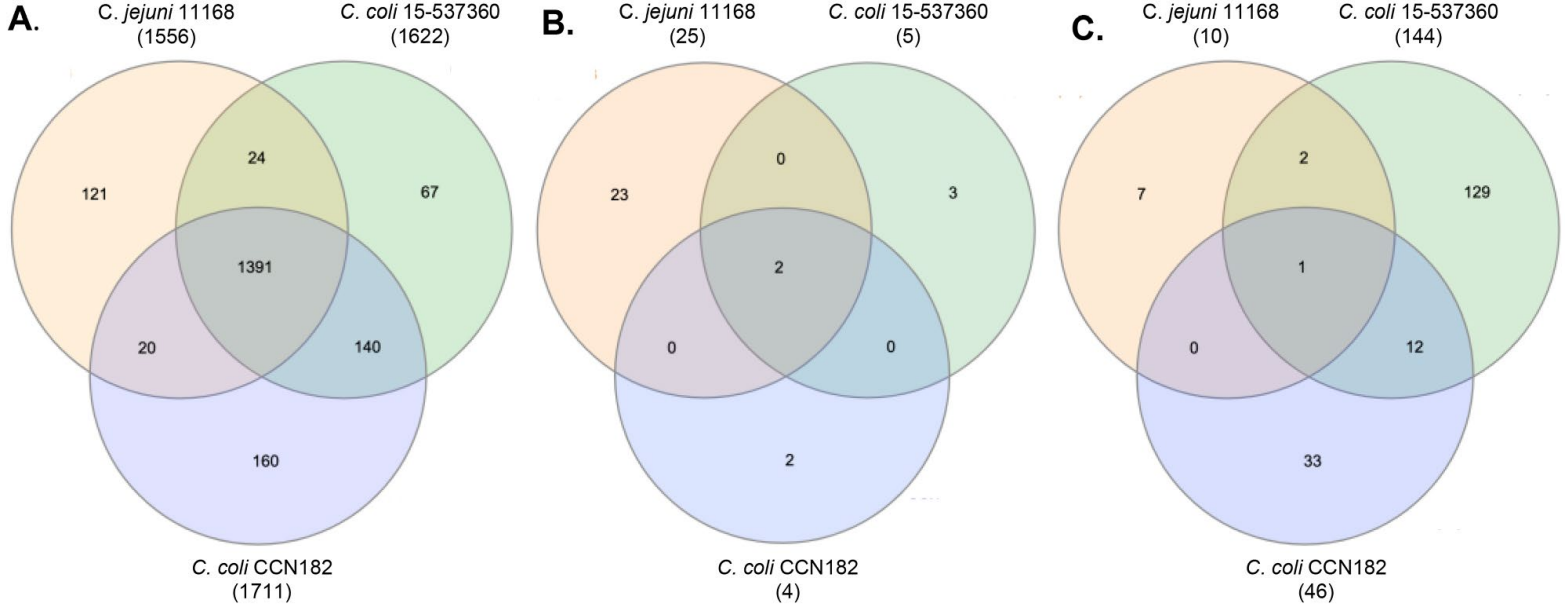
Venn diagrams of the number of overlapping genes that were present in the genomes (**A**), over-represented Tn-insertion mutants (**B**) and under-represented Tn-insertion mutants (**C**) across all strains in this study. Over-represented and under-represented Tn-insertion mutants were determined using a Log_2_ fold change of 2 or -2, respectively, and a q value of less than 0.05. Note, the number of genes associated to *C. coli* CCN182 are decreased from the numbers written in Table 2 due to the removal of collapsed gene families with more than one locus tag associated

To compare the response to oxidative stress across the three strains, a pairwise BLASTP analysis was performed followed by the building of a network model [22]. Across all three genomes, 1391 genes were found to be core (present in all three strains tested here), with 140 genes being present only in the two *C. coli* strains (Fig. 2A). The full list of core genes can be found in Supplementary File 2.xls. Using this form of analysis, which groups homologous genes together, it is possible to compare which Tn-insertion mutants were over- or under-represented across all three strains tested in this study. Across all three strains, there were 2 genes whose disruption caused an advantage under H_2_O_2_, these were *perR* and *ahpC* (Fig. 2B), whilst there was only 1 gene whose disruption caused a detrimental response to oxidative stress which was *czcD* (Fig. 2C).

There were no further advantageous Tn-insertion mutants in homologous genes that were found to be common in the response to oxidative stress between any two strains. However, there were two disadvantageous Tn-insertion mutants that were found in both *C. jejuni* 11168 and *C. coli* 15-537360, which were a bacterioferritin (Cj1534c) and *tpx* (thiol peroxidase). Between the two *C. coli* strains there were 12 under-represented Tn-insertion mutants that were common (Table 2). These included two NAD(P)H-dependent oxidoreductases, one of which is functional (N149_1003) and one of which is the pseudogene NAD(P)H-dependent oxidoreductase (N149_1002). Interestingly in *C. coli* CCN182, the homologous gene to N149_1002 is not annotated as a pseudogene using Bakta, which is able to detect pseudogenes [27]. Transposon insertions in two hypothetical proteins were found to be disadvantageous in both *C. coli* strains under oxidative stress (N149_0828 and N149_0156). Interpro searches found that the hypothetical protein, N149_0828, has homologous regions to the CreD inner membrane family, whilst N149_0156 has no homologous regions to any family but has several transmembrane regions predicted [25].

**Table 2:** Genes and protein products in which Tn-insertion mutants caused a disadvantageous effect in both *C. coli* 15-537360 and *C. coli* CCN182 under oxidative stress

Locus tag in C. coli 15-537360	Protein Genbank ID	Product
N149_0156	AGZ20640.1	hypothetical protein
N149_0679	AGZ21141	molybdopterin adenylyltransferase
N149_0712	AGZ21168.1	methionine ABC transporter substrate-binding protein
N149_0828	AGZ21281.1	hypothetical protein
N149_0863	AGZ21306.1	ABC-F family ATP-binding cassette domain-containing protein
N149_0914	AGZ21356.1	PaaI family thioesterase
N149_0916	AGZ21358.1	thermonuclease family protein
N149_1002		Pseudogene (NAD(P)H-dependent oxidoreductase)
N149_1003	AGZ21440.1	NAD(P)H-dependent oxidoreductase
N149_1256	AGZ21682.2	DUF4910 domain-containing protein
N149_1366	AGZ21784.1	ribosome silencing factor
N149_1501	AGZ21915.1	serine hydrolase family protein

Table shows locus tags in *C. coli* 15-537360 as this is the better annotated strain

### Confirming the role of PerR and CzcD in oxidative stress response

We selected two genes to validate the TraDIS results. We chose two genes that were found in all three strains, one whose mutation was found to be advantageous under H_2_O_2_ (*perR)* and one that whose mutation was found to be disadvantageous (*czcD)*. The role of PerR has previously been described for *C. jejuni* 11168 however, to the best of our knowledge, has not been defined for *C. coli* [8]. Based on a literature search, the role of CzcD in oxidative stress response has not been described before in any *Campylobacter* species. The nature of TraDIS experiments means that these genes have not only been tested across multiple strains but also for multiple mutants within the TraDIS pool. For example, for *perR*, there were (on average across each set of three replicates) 19 unique Tn mutants in *C. jejuni* 11168, 11 unique Tn mutants in *C. coli* 15-537360 and 23 unique Tn mutants in *C. coli* CCN182 tested within these experiments. Similarly, for *czcD*, there were (on average) 21 unique Tn mutants in *C. jejuni* 11168, 11 unique Tn mutants in *C. coli* 15-537360 and 53 unique Tn mutants in *C. coli* CCN182. To confirm the role in *C. coli*, *perR* and *czcD* were deleted using allelic replacement with a chloramphenicol acetyltransferase *cat* resistance cassette in the *C. coli* 15-537360 strain. For each defined mutant produced, each was then complemented through insertion of the gene into a pseudogene (N149_01900). Both genes were placed under the control of a chloramphenicol acetyltransferase *cat* resistance promoter. The deletions and complementation strains underwent H_2_O_2_ exposure for 16 h, under identical conditions to the Tn-insertion mutant libraries alongside the wild-type strain. Validation in just one strain was chosen as the overall number of unique Tn mutants across all three strains (e.g. overall 53 unique Tn mutants in *perR* and 85 unique Tn mutants in *czcD)* provided strong evidence alone that these genes play a role in the oxidative stress response.

**Fig. 3:**
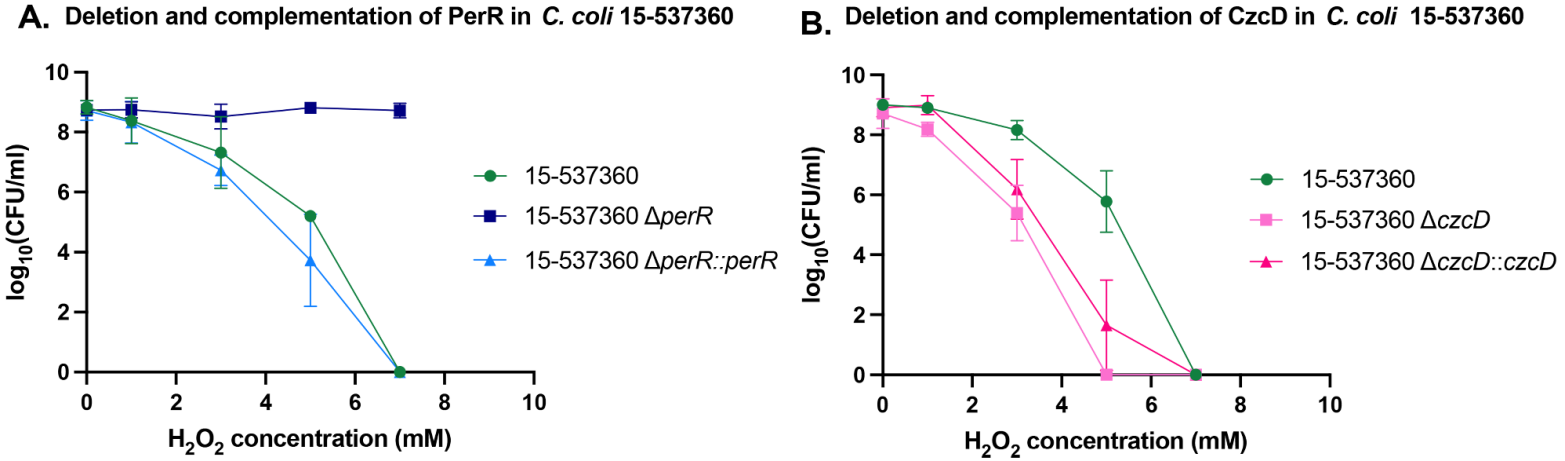
Survival of *C. coli* 15-537360 deletion and complement strains after exposure to various concentrations of H_2_O_2_. The mean Log_10_(CFU/mL) and standard deviation plotted against increasing H_2_O_2_ concentrations after incubation for 16 h (*N*≥3). Graphs show the effect of deletion and complementation of *perR* (**A**) and *czcD* (**B**) after exposure to varying levels of H_2_O_2_

The effect of deleting *perR* in *C. coli* 15-537360 showed an advantageous effect under H_2_O_2_ concentrations (Fig. 3A). For *C. coli* 15-537360 Δ*perR*, there was no decrease in survival over the increasing concentrations of H_2_O_2_, whereas the wild-type and complemented strain showed a similar decrease in survival over the increasing concentrations of H_2_O_2_ with no survival at 7mM. The effect of deletion of *czcD* in *C. coli* 15-537360 showed a disadvantageous effect under H_2_O_2_ (Fig. 3B). Compared to the wild-type, there was a decrease in survival, especially seen at 3mM and 5mM H_2_O_2_. Complementation of *czcD*, however, did not revert the phenotype to wild-type levels of survival.

## Discussion

This study investigated two *Campylobacter* spp, the leading cause of bacterial food-poisoning, after exposure to H_2_O_2_ to examine genes required for surviving oxidative stress. This was investigated using TraDIS to assess previously made Tn-insertion mutant library pools in one *C. jejuni* strain and two *C. coli* strains. This study not only looked at the effect within one strain in one species but compared the results across the two *C. coli* strains and one *C. jejuni* strain. Using this comparison, the disruption of 3 genes were found to have a (advantageous or disadvantageous) effect under H_2_O_2_ exposure across all three strains tested. Two of these were taken forward in one strain to validate the results to the TraDIS analysis and confirm that these genes play a role in oxidative stress.

There was a difference in the ability to survive at different concentrations across the two species (Fig. 1A), and a difference in the number of mutants that were detrimental or beneficial to survival (Table 1). Both *C. coli* strains had an ability to withstand higher H_2_O_2_ concentrations, compared with *C. jejuni* 11168 which could not survive past 4mM of H_2_O_2_. These strains all came from different sources and belong to different sequence types [22]. The effect of these differences (possibly due to differences in genes present, and how the proteins interact under stress conditions) is likely to cause this variance in survival at different concentrations of H_2_O_2_. For *C. jejuni* 11168 there was a difference in the survival of the Tn-insertion mutant pool compared to the wild-type (Fig. 1A and B). This could be down to the mutants in the pool competing or compensating under H_2_O_2_ exposure. For each strain, we selected the H_2_O_2_ concentration for TraDIS to provide 60–80% survival of the Tn-insertion mutant library. It is noticeable that the number of Tn-insertion mutants that were significantly under- or over-represented is small, especially in *C. jejuni* 11168. It is possible that this selection of 60–80% survival was too much and killed off too many Tn-insertion mutants thereby losing genes that play minor roles in the oxidative stress response.

Comparison between the Tn-insertion mutants that were advantageous or disadvantageous after H_2_O_2_ exposure were compared across all three strains. Transposon-insertion mutants in the genes that encode PerR and AhpC have been found to play a role in the oxidative stress in *C. jejuni*, however this study indicates that they may have a similar role in *C. coli*. PerR is the peroxide resistance regulator that negatively regulates the transcription of superoxide and peroxide resistance genes. In *C. jejuni*, when inactivated, there is no repression of transcription of other oxidative stress genes whose products encode AhpC, SodB and KatA – all of which are antioxidants and process H_2_O_2_ [24]. In *C. coli* 15-537360, the deletion of *perR* caused the strain to survive to at least 7mM H_2_O_2_. The re-introduction of *perR* into a pseudogene under a chloramphenicol acetyl transferase, *cat*, promoter produced a survival curve similar to the wild-type, with complete death by 7mM H_2_O_2_. These results support the role of PerR as a negative regulator of oxidative stress proteins in *C. coli* as there is a resulting fitness advantage when the gene is disrupted under H_2_O_2_ stress.

AhpC, which was not validated within this study using defined gene-deletion knockout and complementation, was also a gene that overlapped across all three strains, with the Tn-insertion mutant being able to survive under H_2_O_2_ stress. The specific role of the antioxidant, AhpC (alkyl hydroperoxide reductase) is a peroxiredoxin that converts H_2_O_2_ into water and alcohol. The inactivation of the *ahpC* gene has previously been found to have a compensatory effect when using H_2_O_2_ [8]. Palyada et al., hypothesised that in the absence of the major scavenger of H_2_O_2_, this would lead to an increase in the cytoplasmic H_2_O_2_ levels and therefore activate a further oxidative stress response, resulting in expression of *katA* or *tpx* and *bcp* [8].

Intriguingly, the only Tn-insertion mutants that were detrimental to survival under oxidative stress conditions across all three strains were in *czcD*. The gene *czcD* encodes a cation diffusion protein that functions as a Zinc (Zn(II)) exporter. The translation of this gene is controlled independently by zinc itself, and by an upstream RNA thermometer [28]. Translation is inhibited by this upstream RNA thermometer at temperatures below 37 °C [28]. Alongside this, 2 previous TraDIS studies found that Tn-insertion mutants in *czcD*, significantly affected the ability of the mutants to colonize murine intestine and survive in chicken juice at low temperatures [29, 30]. It has been hypothesised that CzcD plays a role in overcoming the zinc toxicity mediated by the innate immune system in hosts using metal and temperature regulation [28]. The role of CzcD has not previously been linked to the oxidative stress response. However, the link between metal toxicity and oxidative stress has been found before in bacteria, including in *C. jejuni*. In *E. coli*, the oxidative stress response happens under many conditions, including heavy metals [31], whilst in *C. jejuni* it has been found that disruption of copper transporters, leads to the bacteria being more susceptible to oxidative stress due to disruption of copper homeostasis [32]. In this study, the disruption of a zinc transporter, *zupT*, also was disadvantageous under oxidative stress conditions. The deletion of *czcD* in *C. coli* 15-537360 led to a decrease in the ability to survive under the same H_2_O_2_ concentrations as the wild-type, particularly at higher concentrations (3mM onwards), confirming the TraDIS results and that potentially it does play a role in oxidative stress. However, we were unable to complement this by the re-introduction of *czcD* in a pseudogene. This may be due to a variety of reasons, including but not limited to the fact that the native promoter wasn’t known and therefore not used, a polar effect of the complementation technique or the lack of the native genome architecture surrounding *czcD* used - all of which may have an effect and therefore the levels of CzcD produced were unable to produce enough (or produced too much) CzcD. Despite the effect of the deletion of *czcD* was apparent in all three strains and indicates a potential role, investigations will be necessary to fully characterize the role of *czcD* in the oxidative stress response.

Interestingly, after H_2_O_2_ exposure, Tn-insertion mutants in pseudogenes were identified as having a significant advantageous or a disadvantageous effect. These included Tn-insertion mutants in Cj0072c in *C. jejuni* 11168 whose disruption had an advantageous effect and Tn-insertion mutants in three pseudogenes in *C. coli* 15-537360 (N149_1002, N149_1740 and N149_1175) whose disruption had a disadvantageous effect. To confirm that these were indeed pseudogenes at the DNA level, we designed primer pairs to PCR amplify each of these pseudogenes from each strain and then performed Sanger sequencing with each PCR product to confirm the sequence was as expected and had not ‘mutated’ to be able to form a full-length protein. The sequencing confirmed that the genes were still pseudogenes and could not form a full-length protein. Despite confirming this at the DNA level, interestingly the annotated functions of these pseudogenes have links to oxidative stress survival. The *C. jejuni* 11168 pseudogene Cj0072c encodes a putative iron-binding protein, and the Tn-insertion mutant had an advantageous effect on the survival of the bacteria when exposed to H_2_O_2_. Iron and the oxidative stress response are closely linked in *Campylobacter*. Iron (with oxygen) can mediate the generation of reactive oxygen species. Tn mutants in functional genes in iron acquisition and storage were also found to have an increased and decreased oxidative stress tolerance in this study. For *C. jejuni* 11168, Tn mutants in *cft* (ferritin), *cfbpA* (iron-uptake ABC transporter substrate binding protein and *cfbpB* (iron-uptake ABC transporter permease) were found to have an increased tolerance to H_2_O_2_. The deletion of *cft* in the strain *C. jejuni* 81–176 lead to an increased sensitivity to H_2_O_2_, rather than increased tolerance [33]. The reason for this difference is unclear but could be due to different strains, a difference in H_2_O_2_ assay or the way in which the mutants were constructed. In *C. coli*, 3 pseudogenes were found to have a disadvantageous effect under H_2_O_2_ stress. N149_01175 is annotated as an AgrC accessory regulator, which in other bacteria (such as *Staphylococcus aureus*) has been found to be the sensor kinase of the Agr quorum sensing system that mediates bacterial oxidation [26]. N149_01740 is annotated as a bacteriohemerythrin. In *C. jejuni*, other hemerythrin’s have been found to protect iron-sulphur enzymes from oxidative damage [23]. N149_1002 is annotated as a NAD(P)H-dependent oxidoreductase. Its homolog in *C. coli* CCN182 is not annotated as a pseudogene but codes for a functional protein. In *C. coli* CCN182 the Tn-insertion mutants in this gene lead to a similar detrimental effect under H_2_O_2_ stress. Without protein level analysis, we are unable to conclude if these pseudogenes can code for functional proteins, however recently it has been found in other bacteria (such as *Salmonella enterica)*, that many pseudogenes are still disrupted at the mRNA level but there was successful translation of pseudogenes at a protein level [34]. If this is true across other bacteria, these pseudogenes may indeed be functional under oxidative stress and therefore warrant further investigation.

## Conclusions

This study took three Tn-insertion mutant libraries covering one strain of *C. jejuni* and two strains of *C. coli* and investigated the response to H_2_O_2_. To the best of our knowledge this the first published study of a gene fitness analysis of *C. coli* strains and the first to compare between *C. jejuni* and *C. coli* response to H_2_O_2_. The disruption of two genes in all three strains were found to be advantageous (*per* and *ahpC*), this was validated by the deletion and complementation in *C. coli* 15-537360 for *perR* (note - we did not attempt *ahpC*). This finding has also been found previously in *C. jejuni* 11168 and suggests that *perR* works in a similar way in *C. coli.* One Tn-insertion mutant was found to be disadvantageous across all three strains. The role of *czcD* in oxidative stress is novel but the link between other metals and oxidative stress in *C. jejuni* is not. Deletion of *czcD* validated the TraDIS results, however we were unable to complement the phenotype, warranting further investigation into the role of *czcD* under oxidative stress.

## Materials and methods

### Bacterial strains and growth conditions

Three strains across two species were used in this study, one *C. jejuni* strain (11168 [35]) and two *C. coli* strains (15-537360 [36] and CCN182 [37]). *Campylobacter* strains were routinely grown at 42 °C using Mueller-Hinton (MH) agar (Merck) or broth (Oxoid) under microaerobic conditions (5% O_2_, 5% CO_2_, 5% H_2_, 85% N_2_) provided by a Whitley M95 workstation (Don Whitley). All strains and Tn-insertion mutant libraries were stored at -80 °C in Microbank bacterial preservation tubes (ProLabs) until use.

### H_2_O_2_ survival assay using wild-type strains

Wild-type strains were grown in the conditions described above for 48 h, followed by 16 h growth after re-streaking onto fresh agar plates. The bacteria were then harvested from the agar plate using 1 ml of MH broth and diluted to 0.1 OD_600nm_ in 5mls of MH broth. H_2_O_2_ was freshly prepared by diluting the stock solution (Merck, 30% H_2_O_2_) using distilled water and added immediately to each bacterial culture. These liquid cultures were then grown in 50 ml Falcon tubes (with the lids loosened) for 16 h shaking at 200 rpm under conditions described above. A control was also grown with an addition of molecular grade water to the culture. Bacterial growth was measured by determining the CFU/mL before and after the 16-hour incubation. Log_10_(CFU/mL) was plotted against the varying H_2_O_2_ concentrations to fit the Gompertz MIC (Minimal Inhibitory Concentrations) model using Graphpad [38].

### H_2_O_2_ survival assay using Tn-insertion mutant libraries

Creation and analysis of the Tn-insertion mutant libraries within these stains have previously been documented [22] however are briefly detailed here. The *C. jejuni* 11168 Tn library consists of 79,301 unique Tn mutants, giving a rough density of a Tn insertion every 21 bp. There were 522 genes were not disrupted by a Tn insertion. The *C. coli* 15-537360 Tn library has 36,556 unique Tn mutants, meaning the transposon inserted roughly every 45 bp. There were 484 genes that were not disrupted by a Tn insertion. Finally, *C. coli* CCN182 had 104,979 unique Tn mutants, with the Tn roughly inserting every 17 bp. There were 681 genes that were not disrupted by a Tn insertion. The H_2_O_2_ survival assay was the same as described above with the following modifications. For each biological replicate, 0.5 ml of Tn-insertion mutant library stocks were thawed to room temperature and spread directly onto five MH agar plates. Plates were incubated for 48 h under microaerobic conditions. Mutants were harvested using 2 ml MH broth and diluted to 0.1 OD_600nm_. Depending on the strain, this roughly equated to an input of above 2 × 10 [8] CFU/ml, with each input mutant being represented (at least) 1000 times. The appropriate concentration of H_2_O_2_ was added to each culture. A control was also performed, with water being added to the Tn library instead of H_2_O_2_. After 16 h incubation, enumeration of surviving bacteria was performed, and the remaining culture was plated on to agar plates and incubated for a further 48 h. This step was performed to grow out those Tn-insertion mutants that survive the assay and to dilute any DNA originating from any dead bacterial cells. The out-grown Tn-insertion mutants were resuspended and 1 ml of 2 OD_600nm_ was centrifuged, the supernatant was removed and stored at -20 °C. Three biological replicates were performed, each with a control. The Tn library control output (exposed to only water) was compared to the outputs of the Tn libraries exposed to H_2_O_2_ for analysis as documented below.

### DNA isolation and TraDIS

Bacterial cell pellets (including the input and the control) were thawed to room temperature and genomic DNA extracted using Qiagen Genomic tip kit according to the manufacturer’s instructions. The DNA was precipitated using isopropanol and DNA eluted into 50 µl molecular grade water. DNA was quantified using Qubit dsDNA BR assay kit (Invitrogen) and diluted to ~ 25ng/µl. Sequencing libraries were prepared according to Stoakes et al. [22] and sequenced on an Illumina NextSeq 500 using a 500/550 high output V2 kit (75 cycles) (Illumina).

### Analysis of TraDIS sequencing data

Any sequencing reads that did not contain the *mariner* Tn tag (“GGG…(16 bases)…GTT”) were removed using an in-house perl script. The remaining reads were filtered for reads that were longer then 40 bp and any reads not containing at least 40 bp were removed using Cutadapt [39]. The reads were then processed using the Bio-Tradis pipeline [40]. The trimmed and filtered reads from each strain were mapped to their respective genomes using Smalt (*C. jejuni* 11168 – NCBI: NC_002163.10), *C. coli* 15_537360 (GenBank: CP0066703.1) and *C. coli* CCN182 [22]. Briefly, *C. coli* CCN182 was annotated using Bakta v1.6.1 and its Database v4.0 [27]. The tradis_comparison.R script was used to compare each strain assayed with H_2_O_2_ to the control. Mutated genes with significantly decreased Tn-insertion mutant frequency (Log_2_ fold change of less then − 2 and q value less than 0.05) within the H_2_O_2_ library when compared to the control were considered to have a decreased fitness under H_2_O_2_ treatment. Mutated genes with a significantly increased Tn-insertion mutant frequency (Log_2_ fold change of more than 2 and q value less than 0.05) were gain-of-function mutations under H_2_O_2_ compared to the control.

### Comparison of genes across strains

To conduct comparisons across the three strains used, homologous genes across strains needed to be identified. For comparison between the *C. coli* strains and *C. jejuni* 11168, the two-step process using pairwise nucleotide BLAST comparisons followed by a network model, was followed according to Stoakes et al. [22]. . In this analysis, the genes with a high homology for the Bakta annotated *C. coli* CCN182 collapsed into one gene family. Groups with more than one sequence ID associated were removed to avoid genes not actually associated with H_2_O_2_ being considered. This more stringent approach means that some genes associated with H_2_O_2_ will be lost, but there should be no false positives.

### Creation of defined and complemented mutants

To delete the *perR* and *czcD* genes in *C. coli* 15-537360, deletion constructs were designed *in silico* and ordered from GeneArt (ThermoFisher Scientific). The deletion constructs were designed to contain the chloramphenicol acetyltransferase *cat* resistance cassette surrounded by ~ 500 bp upstream and downstream of DNA that normally flanks the gene targeted for deletion.

For complementation of the deleted gene, it was necessary to find a suitable pseudogene to insert the complemented gene. Using *C. coli* 15_537360 all pseudogenes were checked for suitable length (300-400 bp homologous arms) and for a unique enough sequence to not interrupt other genes. The remaining pseudogenes were checked against the *C. coli* BLASTN database to ensure they were pseudogenes in other *C. coli.* N149_01900 which is a frameshifted pseudogene that is annotated as a flagellar basal body protein, FliL satisfied the criteria for selection. A complementation plasmid was synthesized with the same design to the *C. jejuni* complementation plasmid pSV009 [41]. Both *C. coli* genes were put under the control of the chloramphenicol acetyltransferase *cat* resistance promoter. Genes were amplified from the respective genomes and cloned into the PstI and BamHI sites.

Both deletion and complementation plasmids were methylated before natural transformation into *Campylobacter* strains using EcoRI methyltransferase (NEB). Deletion and complementation strains were prepared according to methods described previously [41].

### Confirming the role of genes under H_2_O_2_ exposure

The deletion *perR* and *czcD Campylobacter* strains, their respective complement and the original wild-type strains underwent the H_2_O_2_ survival assay (‘H_2_O_2_ survival assay using wild-type strains’) to confirm their roles.

### Checking significant Tn-insertion mutants identified in pseudogenes

There were four pseudogenes across two strains with Tn insertions that were shown to be significant under H_2_O_2_ exposure. To investigate these further, plot files (which show the location of the Tn insertions) outputted from the BioTradis pipeline, were manually inspected to see if the Tn insertions were distributed across the entire pseudogene, and if the number of reads for each Tn insertion increased (if advantageous) or decreased (if disadvantageous) after H_2_O_2_ stress. If confirmed, the pseudogenes were checked by Sanger sequencing to confirm if they were indeed pseudogenes in our strains.

### Electronic supplementary material

Below is the link to the electronic supplementary material.

Supplementary Material 1

Supplementary Material 2

## Data Availability

The data that support the findings of this study are openly available in EBI ArrayExpress at https://www.ebi.ac.uk/biostudies/arrayexpress/studies/E-MTAB-13336, reference number E-MTAB-13336.

## Abbreviations

ROS: Reactive Oxygen Species
Tn: Transposon
TraDIS: Transposon Directed Insertion-site Sequencing
